# Multifocal Atraumatic Convexity Subarachnoid Hemorrhage

**DOI:** 10.7759/cureus.16091

**Published:** 2021-07-01

**Authors:** Katarina B Dakay, Idrees Azher, Ali Mahta, Karen Furie, Shadi Yaghi, Shawna M Cutting

**Affiliations:** 1 Neurosurgery, Westchester Medical Center, Valhalla, USA; 2 Neurology, University of Texas Health Science Center at Houston, Houston, USA; 3 Neurology, Rhode Island Hospital, Brown University, Providence, USA

**Keywords:** subarachnoid hemorrhage, convexity subarachnoid hemorrhage, mri- magnetic resonance imaging, reversible cerebral vasoconstriction syndrome, cerebral amyloid angiopathy

## Abstract

Background

Multifocal convexity subarachnoid hemorrhage (cSAH) has generally been described in the setting of traumatic brain injury, however, it has also been reported in the absence of trauma in conditions such as with reversible cerebral vasoconstriction syndrome. We describe the clinical and radiographic characteristics of multifocal cSAH in an academic center.

Methods

We analyzed our single-center retrospective database of nontraumatic convexity subarachnoid hemorrhage from January 2015-January 2018. Convexity subarachnoid hemorrhage was defined as blood in one or more cortical sulci in the absence of trauma; patients with blood in the cisterns or Sylvian fissure were excluded. Multifocal location was defined as at least two distinct foci of hemorrhage occurring in two or more lobes. Clinical and neuroimaging data were collected.

Results

Out of 70 total patients with convexity subarachnoid hemorrhage, 13 cases were of multifocal convexity subarachnoid hemorrhage, occurring in 18.6% of all cases. The mean age was 58 years (SD = 14.7). Eleven patients were female. Seven patients had reversible cerebral vasoconstriction syndrome (RCVS)/posterior reversible encephalopathy syndrome (PRES), two had cerebral amyloid angiopathy (CAA), three had intrinsic coagulopathy, and one patient had endocarditis as the etiology of multifocal cSAH. Headache was the most common complaint, in eight (61.5%) patients.

Conclusion

Multifocal cSAH occurs in approximately 18.6% of all cSAH and can occur in the absence of trauma. In our larger cohort of all cSAH, CAA was the most common cause; however, multifocal cSAH is more commonly caused by RCVS/PRES spectrum. Clinicians should be aware that multifocal cSAH can occur in the absence of trauma, and may be a harbinger of RCVS/PRES, particularly in young patients with thunderclap headaches.

## Introduction

Convexity subarachnoid hemorrhage (cSAH) is commonly seen in the context of trauma; however, atraumatic cSAH and accounts for approximately 5-7% of spontaneous subarachnoid hemorrhage (SAH). cSAH is defined as hemorrhage in one or more cortical sulci, without extension into the Sylvian fissure or basal cisterns. Unlike diffuse patterns of SAH involving the basal cisterns or Sylvian fissure and are often attributed to aneurysmal rupture, cSAH is most often related or caused by other conditions such as reversible cerebral vasoconstriction (RCVS)/posterior reversible encephalopathy syndrome (PRES), cerebral amyloid angiopathy (CAA), or venous sinus thrombosis.

In many cases, cSAH is focal in nature, and predominantly involves one sulcus; however, multifocal cSAHs have been reported previously. Multifocal cSAH is a commonly seen pattern in the context of trauma, occurring in 26.5% of cases [1]; however, multifocal cSAH also can be seen in a minority of atraumatic cSAH. Prior reports of atraumatic multifocal cSAH vary widely by case series when reported, ranging from 8-50%, but the significance of multifocal cSAH on ascertaining the etiology of cSAH has not been previously studied to our knowledge [2-6]. We hypothesized based on prior experience that multifocal cSAH may be suggestive of a diffuse process such as RCVS or coagulopathy, and less likely seen in structural causes of cSAH such as venous sinus thrombosis or tumor. In this study, we sought to compare the clinico-radiographic features of atraumatic cSAH cases from a single academic center to what has been reported in the literature.

This article was previously presented as a meeting abstract at the 2020 American Academy of Neurology Annual Meeting on April 14, 2020.

## Materials and methods

This is a retrospective series of consecutive patients who were admitted to a single academic comprehensive stroke center from January 2015 to January 2018. Inclusion criteria were patients 18 or older with an atraumatic, multifocal convexity subarachnoid hemorrhage admitted to our academic comprehensive stroke center from January 2015 to January 2018. We queried our cSAH database which included 70 patients with atraumatic cSAH. cSAH was defined as hemorrhage in one or more cortical sulci, without extension into the Sylvian fissure, or basal cisterns. Patients with a clear antecedent history of trauma, aneurysmal pattern SAH, and those with a clear structural lesion causing the cSAH seen on computerized tomography scan (CT) such as tumor or stroke, were excluded. Multifocal cSAH was defined as cortical subarachnoid hemorrhage in more than one lobe of the brain, or bilateral cSAH, seen on either CT or magnetic resonance imaging (MRI).

Demographic information, including age, sex, medical comorbidities, clinical testing, and other data related to standard clinical stroke care, was collected as part of an institutional quality improvement project. Imaging data including brain MRI, magnetic resonance angiography (MRA), computerized tomography angiography (CTA), magnetic resonance venogram (MRV), and catheter-based angiogram were collected; all neuroimaging was interpreted by a board-certified, fellowship-trained neuroradiologist. MRI and MRA were performed on a 1.5 T magnet. The etiology of the cSAH was determined based on available clinical and radiographic information, by either a board-certified vascular neurologist or board-certified neurointensivist. Data were abstracted from the electronic medical record into a secure password-protected database (REDCap, Vanderbilt University, Nashville, USA) [7]. This study was approved by the Institutional Review Board; de-identified data is available upon request.

## Results

**Figure 1:**
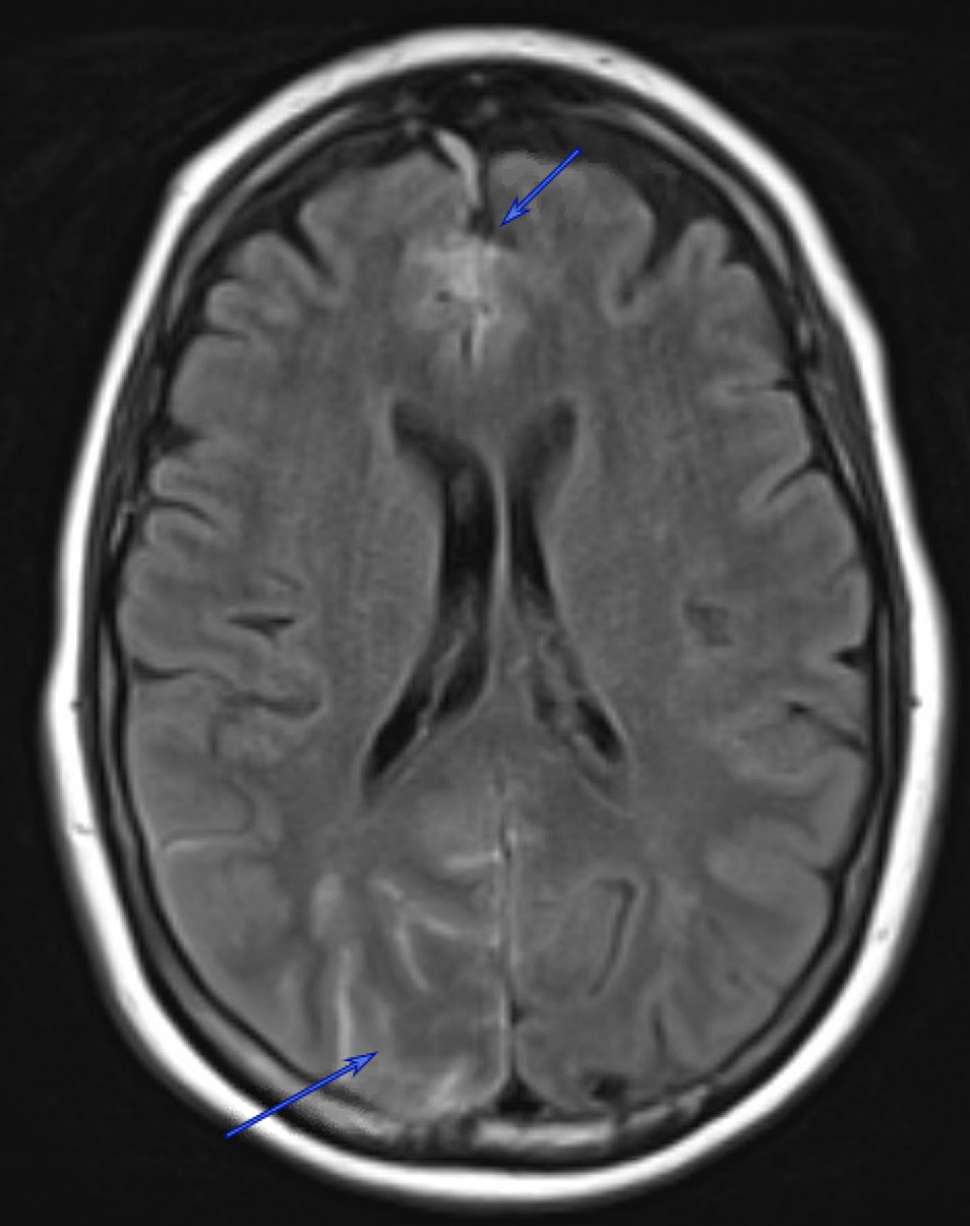
Multifocal Convexity Subarachnoid Hemorrhage Fluid-attenuated inversion recovery (FLAIR) sequence demonstrates two distinctive areas of subarachnoid hemorrhage (blue arrows).

**Figure 2:**
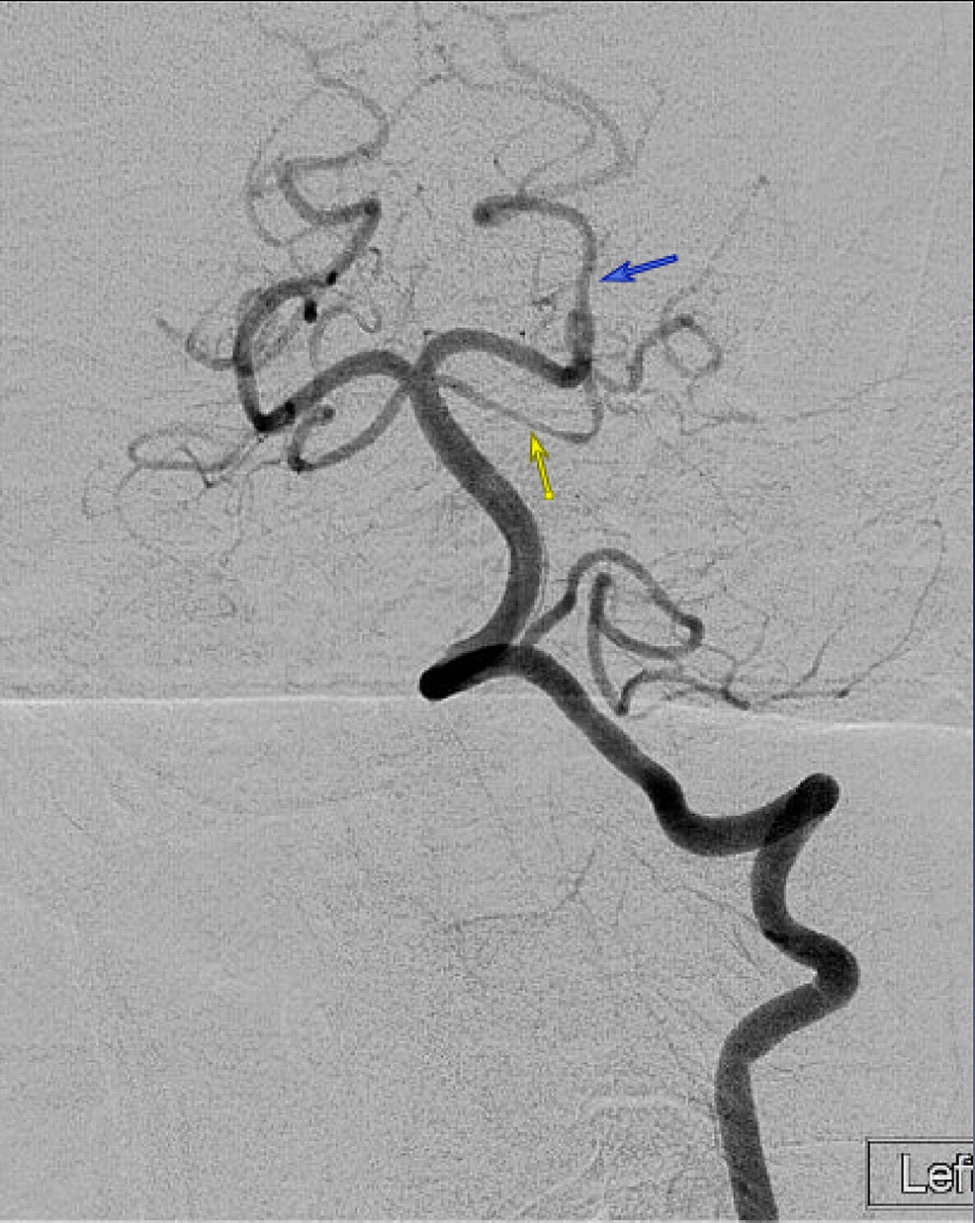
Angiography Angiography in a patient with RCVS demonstrates vasoconstriction in the left superior cerebellar artery (yellow arrow) and left posterior cerebral artery (blue arrow). RCVS=reversible cerebral vasoconstriction syndrome

Of 70 patients with cSAH, a total of 13 patients were identified who had multifocal cSAH; 11 (84.6%) were female and the average age was 58.6 years (range 22-83). All patients underwent CT brain; MRI was performed in a majority of patients. The parietal lobe was the most common region involved. Details of each patient are outlined in Table 1. RCVS/PRES was responsible for most cases, occurring in seven patients (53.8%); CAA accounted for a minority of patients, accounting for only two cases (15.4%). An example of multifocal cSAH induced by RCVS is shown in Figure 1; Figure 2 demonstrates an example of angiographic vasoconstriction seen in RCVS. The remainder of the cases were explained by coagulopathy in three cases, and one isolated case of mycotic aneurysm associated with endocarditis. For all cases of RCVS/PRES, a potential inciting factor was identified including hypertension in one patient, prescription or over-the-counter vasoactive substances in three patients, recreational substances in one patient, and two cases of circumstantial triggers including Valsalva and extreme emotional stress.

**Table 1:** Case Series cSAH=convexity subarachnoid hemorrhage; RCVS=reversible cerebral vasoconstriction syndrome; CAA=cerebral amyloid angiopathy; mRS=modified Rankin scale; PRES=posterior reversible encephalopathy syndrome

Patient	Age	Sex	Medical Comorbidities	Clinical Presentation	Onset to Imaging (days)	Location of cSAH	Etiology of cSAH	Precipitating or Aggravating Factors (if known)	Outcome
Patient 1	65	F	Hypertension, diabetes	Thunderclap headache; confusion	1	Bilateral parietal and occipital lobes; interhemispheric fissure	RCVS	Serotonin-norepinephrine reuptake inhibitor	Discharged home; mRS of 1 at 90d
Patient 2	54	F	Hyperlipidemia	Thunderclap headache with nuchal rigidity	0	Bilateral parietal lobes	RCVS	Valsalva	Discharged home; lost to follow-up
Patient 3	73	F	Hypertension, diabetes, dementia, coronary artery disease, atrial fibrillation (not anticoagulated)	Altered mental status; lethargy and difficulty walking	4	Parietal and frontal lobes	CAA	N/A	Readmission for urinary infection; lost to follow-up
Patient 4	49	F	Migraine, diabetes, COPD	Thunderclap headache	7	Bilateral parietal and occipital lobes	RCVS	Decongestant use	Discharged home; mRS of 0 at 90 days, returned to work
Patient 5	58	M	Diabetes, hyperlipidemia, renal and hepatic disease	Thunderclap headache; diffuse numbness and tremor	6	Right frontal and left parietal	Thrombocytopenia due to cirrhosis	Aspirin	mRS of 1 at 90 days
Patient 6	57	F	Cancer, migraine, hypertension, COPD	Thunderclap headache	0	Multifocal	RCVS	Emotional stress/heated argument	mRS 0 at 90 days
Patient 7	83	M	Hypertension, diabetes, atrial fibrillation	Left arm tingling and epistaxis	1	Multifocal	Aplastic anemia	Aspirin	Lost to follow-up
Patient 8	22	F	Prior aneurysmal subarachnoid hemorrhage	Abdominal pain and fever	2-3	Frontal, parietal and occipital lobes	Endocarditis	N/A	Lost to follow-up
Patient 9	75	F	Coronary artery disease, renal disease	Altered mental status after knee surgery	4	Frontal and parietal lobes	CAA	Prophylactic dose lovenox	Discharged to rehab; mRS 3 at 90 days
Patient 10	55	F	Acute lymphocytic leukemia, idiopathic thrombocytopenic purpura, hyperlipidemia	Headache; known leukemia	2	Multifocal	Thrombocytopenia due to idiopathic thrombocytopenic purpura	N/A	Transferred to another hospital; lost to follow-up
Patient 11	63	F	Hypertension, epilepsy	Confusion, blurry vision, headache with nausea/vomiting	0	Multifocal	PRES	Hypertension	Readmitted within 90 days for hypertensive urgency; mRS 1 at 90 days
Patient 12	52	F	Hypertension	Severe headache with nausea/vomiting; scotomas	3	Interhemispheric fissure, parietal	RCVS	Marijuana	mRS of 2 at 90 days
Patient 13	56	F	Hypertension, prior stroke, prior breast cancer	Severe headache, weakness	0	Multifocal	RCVS	Selective serotonin reuptake inhibitor	Developed subsequent intracerebral hemorrhage requiring decompressive hemicranectomy; mRS of 4 at 90 days

Compared to our larger cohort [8], several differences were noted. First, RCVS was more commonly implicated in multifocal cSAH, accounting for 53.8% of cases of multifocal cSAH and only 20% of the larger cohort. CAA was the most common etiology of our total cSAH cohort, whereas it accounted for a minority of multifocal cSAH cases. In keeping with the higher incidence of CAA, the age was younger in the multifocal cSAH cohort at 58 years, compared to 64.7 in all cases of cSAH. Headache was the most common presenting factor in both cohorts, but was more common in the multifocal cSAH group, occurring in 37% of the all cSAH cohort and 61.5% of the cSAH cohort. Additionally, while there were four cases of venous sinus thrombosis and four cases of tumor in our larger cohort, no cases of either venous sinus thrombosis or tumor presented with multifocal cSAH. 

## Discussion

Multifocal cSAH has often been attributed to trauma, occurs in 26.5% of trauma-associated cSAH, and is likely due to the diffuse impact of closed head injury. Our case series demonstrates that it is also common in atraumatic cSAH, occurring in 18.6% of patients in our series. Other published studies have suggested that the incidence ranges from 8-50% of all cases [2-6]; the frequency in prior case series is presented in Table 2.

**Table 2:** Incidence of Multifocal Convexity Subarachnoid Hemorrhage in Prior Case Series cSAH=convexity subarachnoid hemorrhage; CAA=cerebral amyloid angiopathy; RCVS=reversible cerebral vasoconstriction syndrome; PRES=posterior reversible encephalopathy syndrome; NR=not reported

Author (Year)	Number of total patients	Most common cause of cSAH (N)	Number of multifocal cSAH	N (Percent) of patients with CAA as etiology	N (Percent) w RCVS/PRES as etiology
Spitzer (2005)	12	PRES/RCVS (4)	1 (8%)	0(0%)	4 (25%)
Refai (2008)	20	RCVS/PRES (7)	3 (15%)	0 (0%)	11 (54%)
Kumar (2010)	29	RCVS/PRES (11)	5 (17%)	10 (35%)	11 (38%)
Beitzke (2011)	24	NR	12 (50%)	5 (21%)	NR
Bruno (2013)	34	RCVS/PRES (13)	3 (9%)	13 (38%)	7 (20%)

While the causes of atraumatic cSAH are broad, the multifocal pattern of cSAH may be more likely to occur in diffuse processes such as RCVS or coagulopathy as compared to more focal pathologies as tumors or venous sinus thrombosis. In our larger cohort of 70 patients with cSAH, four patients had venous sinus thrombosis and four had tumors; however, all cases were focal in the region of the lesion, and none presented with multifocal cSAH.

In RCVS, vasoconstriction of the intracranial vessels and subsequent vasodilatation is thought to cause rupture of friable cortical pial vessels leading to cSAH [9-10]; this process affects the distal vessels typically in a diffuse pattern. In fact, cSAH is part of the RCVS2 score, a clinical algorithm designed to differentiate RCVS from other causes of vasculopathy [11] and hemorrhagic complications are known to occur in ⅓ of patients with RCVS [12]. Similarly, it is likely that a systemic coagulopathy predisposing to cSAH would affect the cortical vessels equally without the focality of a structural lesion, increasing the likelihood of multifocal cSAH. This series suggests that the pattern of the cSAH, particularly the presence of cSAH in multiple locations, may aid in diagnosing the underlying etiology.

The limitations of this study inherent to its nature are that it is a single-center, retrospective case series with a relatively small cohort which may not be generalizable. The most common etiologies of cSAH have been shown to vary dramatically from center to center, depending on the average age of the cohort population, medical comorbidities, and other factors. Additionally, subarachnoid hemorrhage is known to redistribute, although most of the patients in our cohort were imaged shortly after the onset of symptoms. Follow-up imaging was not available in some patients. However, this study provides some preliminary data on the incidence of multifocal atraumatic cSAH and demonstrates the potential utility it may have in elucidating the underlying diagnosis. Larger prospective studies are needed to corroborate our results.

## Conclusions

Multifocal cSAH is a commonly seen hemorrhage pattern in trauma but can occur in cases of spontaneous convexity subarachnoid hemorrhage as well. Most cases can be attributed to RCVS, likely due to the diffuse distribution of the vasoconstriction. Multifocal cSAH is less likely to be seen in focal pathologies such as venous sinus thrombosis. Our findings suggest that the pattern of bleeding may help identify the cause of convexity subarachnoid hemorrhage. However, further studies are needed to confirm these results.
